# The effect of cigarette smoking, alcohol consumption and fruit and vegetable consumption on IVF outcomes: a review and presentation of original data

**DOI:** 10.1186/s12958-015-0133-x

**Published:** 2015-12-16

**Authors:** Sarah Firns, Vinicius Fernandes Cruzat, Kevin Noel Keane, Karen A. Joesbury, Andy H. Lee, Philip Newsholme, John L. Yovich

**Affiliations:** School of Biomedical Sciences, CHIRI Biosciences Research Precinct, Curtin University, Kent St, Bentley, Perth, WA 6102 Australia; Department of Epidemiology and Biostatistics, School of Public Health, Curtin University, Kent St, Bentley, Perth, WA 6102 Australia; PIVET Medical Centre, 166-168 Cambridge Street, Leederville, Perth, WA 6007 Australia

## Abstract

**Background:**

Lifestyle factors including cigarette smoking, alcohol consumption and nutritional habits impact on health, wellness, and the risk of chronic diseases. In the areas of *in-vitro* fertilization (IVF) and pregnancy, lifestyle factors influence oocyte production, fertilization rates, pregnancy and pregnancy loss, while chronic, low-grade oxidative stress may underlie poor outcomes for some IVF cases.

**Methods:**

Here, we review the current literature and present some original, previously unpublished data, obtained from couples attending the PIVET Medical Centre in Western Australia.

**Results:**

During the study, 80 % of females and 70 % of male partners completed a 1-week diary documenting their smoking, alcohol and fruit and vegetable intake. The subsequent clinical outcomes of their IVF treatment such as quantity of oocytes collected, fertilization rates, pregnancy and pregnancy loss were submitted to multiple regression analysis, in order to investigate the relationship between patients, treatment and the recorded lifestyle factors. Of significance, it was found that male smoking caused an increased risk of pregnancy loss (*p* = 0.029), while female smoking caused an adverse effect on ovarian reserve. Both alcohol consumption (β = 0.074, *p* < 0.001) and fruit and vegetable consumption (β = 0.034, *p* < 0.001) had positive effects on fertilization.

**Conclusion:**

Based on our results and the current literature, there is an important impact of lifestyle factors on IVF clinical outcomes. Currently, there are conflicting results regarding other lifestyle factors such as nutritional habits and alcohol consumption, but it is apparent that chronic oxidative stress induced by lifestyle factors and poor nutritional habits associate with a lower rate of IVF success.

## Background

*In-vitro* fertilization (IVF) is an assisted reproductive technology (ART) in which the ovum is fertilized by sperm outside of the body. The zygote is cultured in growth medium for approximately 5 days and the resulting embryo or blastocyst is transferred back into the women. The first IVF pregnancy was achieved in 1978 and since then, IVF and its variant intra-cytoplasmic sperm injection (ICSI), has become the main form of ART used to treat infertility for both males and females [1]. The use of ARTs such as IVF is increasing. Therefore, it is highly important to know all factors that can affect the success rate of IVF, especially due to the emotional costs, time and money invested into treatment cycles. One key area of research where data is lacking is how prominent lifestyle factors such as, cigarette smoking, alcohol consumption and adequate ingestion of fruits and vegetables, impact on IVF outcomes.

Several reports have suggested that cigarette smoking [2], alcohol consumption [3, 4] and nutritional intake [5] all impact on natural reproduction and fertility, and thus could be a cause of infertility in some cases, which may contribute to the increase of patients accessing ART. It is entirely possible that changes in lifestyle factors before treatment could lead to a natural restoration of fertility, and could reduce the requirement for ART procedures [6]. Consequently, it is essential to understand how these lifestyle factors may affect IVF outcomes, particularly because they are modifiable behaviours that could potentially be altered to enhance the chance of IVF success.

Clearly, most patients undergoing IVF treatment cycles are willing to modify their lifestyle behaviour, and therefore it is crucial they are aware of the precise lifestyle changes they can implement to enhance the likelihood of IVF success. Hence, the aim of this study was to determine the effect of cigarette smoking, alcohol consumption and nutritional aspects (fruits and vegetable ingestion) on common IVF outcomes including, oocyte production, fertilization rates, β-hCG pregnancy and first-trimester pregnancy loss. In the present work, we review the current, but limited literature available, relating to the impact of several lifestyle factors on reproduction, and the subsequent IVF parameters measured. We also include some previously unpublished data, obtained from a cohort study on couples undertaking IVF treatment at our private IVF clinic, PIVET Medical Centre.

## Materials and methods used to obtain original data

### Subjects and data collection

The original data presented in this article was obtained from a prospective cohort of 351 couples undergoing IVF treatment at PIVET Medical Centre between January 1997 and August 1998. Lifestyle data was recorded using a Lifestyle Questionnaire/Diary, which was used to collect data for years of smoking (smoke years), nicotine, alcohol intake and fruit and vegetable consumption. The diary was completed from day 4 to day 10 of the treatment cycle. Nicotine intake (mg) was measured by the amount of cigarettes smoked by nicotine content. Alcohol intake per week was measured as the number of standard 10 g alcoholic beverages and fruit and vegetable consumption was measured in standard serves. All data collected was self-reported by the patients, with 80 and 70 % of females and males retuning dietary diaries, respectively. Reporting of these data was approved under the Curtin University Ethics Committee approval number RD_25-10, general approval for retrospective data analysis (2011).

### Follicle stimulation and oocyte retrieval

Levels of serum FSH, LH and oestradiol were measured on day 2 of the treatment cycle. Ovarian follicle stimulation in the majority of women was achieved using the ‘flare’ regimen, which involves daily injections from day 2 to around day 6 of the treatment cycle with 10–20 IU/l leuprorelin acetate (Lucrin, Abbott Australasia Pty Ltd, Kurnell, (Australia) to supress pituitary gonanatrophin release. Patients were then stimulated with purified FSH, Metrodin HP (Serono, Aubonne, Switzerland), human menopausal gonadotrophin (hMG), Humegon, or recombinant human FSH (both Organon Australia Pty Ltd). Dosage was specific to each patient dependent on ovarian response monitored by oestradiol concentrations and vaginal sonography. Follicular maturation was triggered by an injection of hCG (10 000 IU; Pregnyl, Organon Australia Pty Ltd). Oocytes were retrieved by Transvaginal oocyte aspiration (TVOA) 36 h after hCG injection using a PIVET-Cook Laparoscopic/Ultrasound Double-Lumen Ovum Pickup Needle (Cook Australia Pty Ltd).

### Sperm preparation and insemination

Ejaculates were obtained 1–2 h after oocyte retrieval. Fertilization was by either conventional in vitro fertilization (IVF), or intra-cytoplasmic sperm injection (ICSI). Fertilization was indicated by the presence of two pronuclei, at 18–20 h post-insemination.

### Embryo quality and transfer

Embryos were graded on a scale from 1 to 4 in increments of 0.5: 4 = symmetrical, spherical blastomeres and no extracellular fragmentation, 3 = symmetrical, spherical blastomeres with low to moderate fragmentation, 2 = irregular blastomeres with moderate to considerable fragmentation, 1 = poorly defined blastomeres and considerable fragmentation [7]. The best quality embryos were selected for transfer. The collective quality of embryos transferred was measured using the modified Cumulative Embryo Score (CES), which is calculated by multiplying the grade of the embryo with number of blastomeres for each embryo selected for transfer, and then adding these values together [8]. The modified CES took into account the claim that rapidly developing embryos implant less frequently [9], embryos with 5, 6, 7 or 8 blastomeres were treated as having 3, 3, 2 or 2 blastomeres respectively. Number of embryos transferred was determined by the number of embryos available, and guidelines by the Western Australia Reproductive Technology Council (WARTC). At the time the research was undertaken, the WARTC specified that a maximum of three embryos could be transferred, with four in exceptional cases, such as advanced age or multiple unsuccessful IVF attempts. However, currently in Australia, single embryos are mostly transferred in order to reduce multiple birth rates and decrease risk of adverse perinatal outcomes. At the time this study and data collection was performed, couples that were deemed to have a high chance of pregnancy had two embryos transferred, even if there were more than two embryos available and were defined as 2-embryo transfer by choice.

### Assessment of pregnancy

Pregnancy was determined by a positive β-hCG pregnancy test 16 days after embryo transfer, defined as β-hCG levels > 25 IU/l and progesterone and oestradiol levels consistent with 4th week of pregnancy. Pregnancy loss in the first trimester was defined as a positive β-hCG test that failed to reach the 12th week.

### Statistical analysis

Data was originally analysed using SPSS package version 11 and recently validated using SPSS version 22. Multiple regression analysis was also undertaken to assess the relationship between lifestyle factors and IVF outcomes.

## Impact of lifestyle factors

### Cigarette smoking

Cigarette smoke contains several toxic chemical compounds known to be mutagens and carcinogens such as cotinine and benzo[a]pyrene [10]. Inhaling cigarette smoke can decrease fertility and may affect reproductive outcomes such as causing delayed conception in active male smoking, in addition to active and passive female smoking [2, 11]. Active and passive smoking also causes increased risk of miscarriage during pregnancy, which is potentiated by the amount of cigarettes smoked per day [12].

**Fig. 1:**
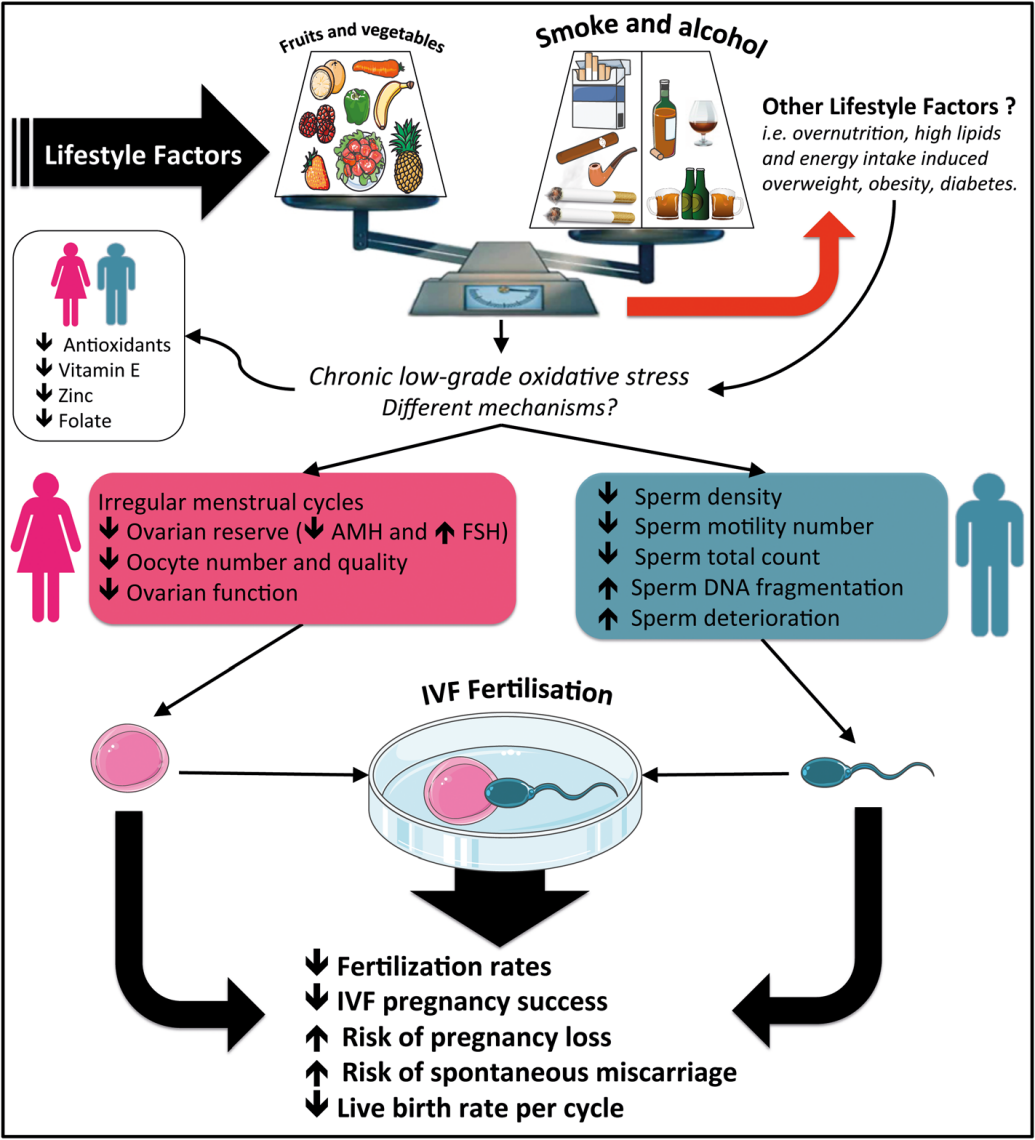
The impact of lifestyle factors on IVF outcomes. From the time of primates, fruits and vegetables consumption were part of the diet and considered an excellent source of essential nutrients, such as vitamins and minerals, among others that support the balance between Reactive Oxygen Species (ROS) and the antioxidant defences. However, modern lifestyle choices, including smoking, alcohol abuse and over-nutrition, can promote chronic low-grade inflammation and oxidative stress in various organs. Taken together, these lifestyle factors may eventually impact on IVF outcomes, having adverse effects on the reproductive system in both, men and women

Smoking may also introduce perturbations in menstrual cycles including promoting shorter or irregular cycles [13], as well as decreasing ovarian reserve, as reflected by lower antral follicle count (AFC) and lower serum anti-Mullerian hormone (AMH) levels (Fig. 1) [14]. Furthermore, cigarette smoking has an impact on age of onset of menopause. In a prospective study, earlier onset of menopause was higher in smoking individuals, when compared to non-smokers, and interestingly, the risk of earlier menopause was decreased in women who had ceased smoking compared to those who were current smokers [15]. Taken together, these studies demonstrate the significant influence of cigarette smoke on several aspects of female fertility.

With regard to male fertility, several studies have demonstrated a significant decrease in sperm density, sperm motility number and total count in smoking individuals (Fig. 1) [4, 16]. More specifically, Künzle et al. [16] found that male cigarette smoking caused a 15.3 % decrease in sperm density, a 17.5 % decrease in total sperm count and a 16.6 % decrease in number of motile sperm. Smoking has also been shown to have an effect on sperm DNA fragmentation (SDF) and other morphological parameters [17]. Using the Halosperm technique, a chromatin dispersion assay that assesses sperm DNA damage, it was shown that heavy smokers had the largest percentage of degenerated sperm and SDF, while non-smoking males had significantly higher semen volumes compared to moderate and heavy smoking males [17].

### Cigarette smoking and IVF

#### PIVET data demonstrating the influence of cigarette smoking on IVF outcomes

Since cigarette smoking appears to have a detrimental effect on fertility, it is reasonable to assume that cigarette smoking may affect the risk or requirement for IVF. Furthermore, it is entirely possible that female smoking during IVF treatment may adversely affect the subsequent IVF outcomes. Cigarette smoking is the most sufficiently researched lifestyle factor that could affect IVF outcomes, compared to nutrient intake and alcohol consumption. However, the literature is still very limited. Original unpublished data from our laboratory examined the effects of cigarette smoking on oocyte production, fertilization rates, pregnancy and pregnancy loss rates. Lifestyle data collected from these patients is presented in Table 1. The mean number of oocytes retrieved from women undergoing IVF treatment did not significantly differ between regular smokers (11.1 oocytes retrieved, SD 6.5), ex-smokers (11.8, SD 10.1) or non-smokers (11.2, SD 7.6) which indicated that smoking did not influence oocyte production.

**Table 1:** Overview of lifestyle factors by gender

		*n*	Min	Max	Mean	SD	Median	IQR
Female								
smoking (years)	Ex-smokers	93	0.5	25.0	8.7	6.4	7.0	10.0
	Occasional smokers	11	5.0	29.0	12.8	6.6	10.0	5.0
	Regular smokers	40	6.0	25.0	15.0	5.7	15.0	10.0
	Never smokers	142	-	-	-	-	-	-
Nicotine (mg/wk)	Occasional smokers	11	0	3.6	1.4	1.3	1.6	2.7
	Regular smokers	40	0.8	175.0	43.5	40.9	31.4	44.6
Alcohol (std drinks/wk)		286	0	30.0	3.1	5.2	0	4.5
F&V (serves/wk)		286	0	67.0	24.9	11.6	24.0	14.8
Male								
Smoke (years)	Ex-smokers	67	0.5	30.0	9.9	6.7	10.0	9.0
	Occasional smokers	11	5.0	25.0	12.8	6.0	11.5	5.8
	Regular smokers	51	0.5	30.0	17.4	6.7	18.0	10.0
	Never smokers	124	-	-	-	-	-	-
Nicotine (mg/wk)	Occasional smokers	11	0	4.1	1.2	1.8	0	3.6
	Regular smokers	51	4.9	245.0	70.7	55.1	59.8	69.6
Alcohol (std drinks/wk)		253	0	79.0	9.9	11.6	6.5	14.0
F&V (serves/wk)		247	0	54.0	21.0	10.6	20.0	14.5

As a mechanism to determine the influence of smoking status on ovarian reserve and as a consequence of the age of the presented data, we utilised basal FSH level as an accepted marker of ovarian reserve rather than AMH in smoking and non-smoking women [18]. It was found that oocyte production lowered along with increased basal FSH levels (*p* < 0.001), and basal FSH appeared to increase in tandem with years of cigarette smoking (β = 0.007, *p* = 0.035), even after adjusting for the effects of age and infertility status. These results suggested a negative relationship was apparent between years of cigarette smoking and oocyte production, although this was not represented in the mean number of oocytes collected between the smoking status groups. However, we speculate that this observation could be related to the rFSH dosage administered during stimulation before oocyte retrieval, which may compensate for any negative effects of cigarette smoke on oocyte production and ovarian function. Interestingly, the results from our laboratory also showed that fertilization rates were not influenced by current female smoking status (Table 2). However, fertilization rates did decrease as female years of smoking increased (*p* < 0.001), and there is an observable and significant interaction between fertilization rates and nicotine (Table 2).

**Table 2:** Multiple logistic regression analysis of *in vitro* fertilisation rates^a,b,c^ (*n* = 152)

Variable	β Coefficient	Standard error	*P*
Constant	0.277	0.214	0.196
Female nicotine (mg/wk)	−0.012	0.009	0.176
Female smoke (years)	−0.047	0.010	< 0.001
Female smoke years*nicotine	0.001	< 0.001	0.044
Male alcohol (std drinks/wk)	0.074	0.014	< 0.001
Male F&V (serves/wk)	0.034	0.007	< 0.001
Male alcohol*F&V	−0.001	0.001	0.011

*depicts the potential interaction between two terms analysed by logistic regression

^a^Y = no. of oocytes fertilised (2PN) weighted by total no. of oocytes retrieved

^b^adjusted R^2^ = 0.236, deviance = 281.508, 141df, *P* < 0.001

^c^non-significant variables: female age, female alcohol, female F&V, male age, oligozoospermia, asthenozoospermia, teratozoospermia, male nicotine and male smoke years

In relation to male patients and applying multiple logistic regression analysis, data from our laboratory showed that male nicotine intake was significantly and positively associated with a first-trimester (up to 12-week) non-ectopic pregnancy loss (*p* = 0.008) (Table 3). The risk of pregnancy loss increased by 2.9 % with every 1 mg of nicotine intake in males and after adjusting for female age, the odds ratio (OR) of a first-trimester pregnancy loss for male smokers was 2.2 (95 % CI 1.1–4.3) when compared to non-smokers (Table 3). We speculate that this association between male smoking and pregnancy loss is likely due to the result of spermatozoal DNA damage that occurred pre-conception. However, this was contradictory to female smoking status, where there was no significant effect on pregnancy loss (Table 3).

**Table 3:** Multiple logistic regression analysis of non-ectopic first trimester pregnancy loss^a,b,c^ (*n* = 68)

Variable	β Coefficient	OR	95 % CI	*P*
Constant	−12.116	-	-	< 0.001
Female age (years)	0.311	1.365	(1.139, 1.636)	< 0.001
Male nicotine (mg/wk)	0.024	1.024	(1.006, 1.042)	0.008

^a^< 12 week pregnancy loss (*n* = 19) and 12 week ongoing pregnancy (*n* = 49)

^b^goodness-of-fit *χ* ^2^ = 18.392, 2df, *P* < 0.001

^c^non-significant variables: embryo quality (mCES), female nicotine, female smoke years, female caffeine, female F&V, male age, male smoke years, male caffeine, male alcohol and male F&V

#### Review of data from the literature demonstrating the impact of cigarette smoking on IVF outcomes

Our original research has demonstrated that cigarette smoking influenced several aspects of fertility and female smoking was associated with increased basal FSH and decreased oocyte fertilization, while male smoking was positively correlated with first-trimester pregnancy loss. A range of other studies have been performed that corroborate some of our findings, while others illustrate the complex interplay between lifestyle factors and positive ART outcomes.

It has been demonstrated that actively smoking women had significantly lower serum AMH, when compared to non-smoking women undergoing to IVF, which indicated a negative impact on ovarian reserve [14]. In addition, it was reported that smoking couples had a reduction in the number of oocytes retrieved (by 40 %), while those with smoking male partners were reduced by a further 5 % [19]. However, other researchers have found that the mean number of oocytes retrieved for smokers (9.6 oocytes) and non-smokers (9.0 oocytes) was not significantly different [20]. Taken together, these outcomes echo those presented in our study above where female smoking was associated with increased basal FSH, but this did not manifest in significant differences in the number of oocytes collected or pregnancy loss rates. Consequently, this observation may be related to rFSH stimulation regimens, where some clinics apply a standard dosage based on female age, while others, including PIVET, applied a step-up dosage for those women not responding optimally [21].

Interestingly, a study conducted in Dutch IVF clinics examined the effect of smoking on the IVF success rate in 8457 women [20], and they showed that smoking decreased the live birth rate by 7.3 %. Furthermore, the spontaneous abortion rate per pregnancy was higher for smokers at 21.4 % compared with 16.4 % for non-smokers [20]. We observed that female smoking years and nicotine intake was not associated with first-trimester pregnancy loss, but the difference between the studies may be first explained in a practical sense by the larger sample size in the Dutch study [20]. Second, there is also the clear possibility that smoking women in our smaller cohort, who have become pregnant following IVF, would alter and probably cease their smoking habits upon being informed of successful pregnancy. It is therefore not astonishing that female smoking status does not have a significant impact on most IVF outcomes in our study as it is likely they will modify their behaviour. However, in other work, the risk ratio (RR) for smoking couples failing to become pregnant was doubled when compared to non-smoking couples [19]. Moreover, the RR increased by 4 % for each additional year of smoking. In addition, the authors reported that live birth rates were significantly decreased for smoking couples in comparison to non-smoking couples [19]. They also demonstrated that if a woman had smoked during her lifetime, she was twice as likely to fail becoming pregnant compared to non-smoking women. This risk eventually increased by 9 % with each additional year of smoking.

A meta-analysis conducted in 2008 included 21 studies that focused on reproductive outcomes and lifestyle factors [22]. They found that cigarette smokers had a significantly lower pregnancy and live birth rates per cycle in comparison with non-smokers. In the same meta-analysis, smoking was also significantly associated with an increased risk of spontaneous miscarriage. However, there was no association between smoking and fertilization rates in 17 studies that included fertilization rate parameters [22]. Conversely, Zitzmann et al. [23] reported that fertilization rates in IVF patients were influenced by female smoking. Importantly, in addition to active smoking status, an association between passive smoking and IVF outcome has also been shown to impair fertility parameters [24]. In one study, patients who were exposed to side stream smoke had lower pregnancy rates that were equal to mainstream smokers [24]. Since other studies have shown that increased levels of reactive oxygen species (ROS) in follicular fluid were correlated with poor oocyte quality [25], there remains the possibility that the decrease in oocyte production may be due to the increased levels of ROS promoted by cigarette smoking over a long period of time. The involvement of ROS in male and female fertility is described in more detail later in the present manuscript. However, despite several laudable studies investigating the effect of female smoking on IVF outcomes, the research is still very limited and much more work is required.

Comparably, the effect of male smoking alone on IVF outcomes is another area that is lacking. It has been indicated that for couples undergoing IVF (*n* = 148) and ICSI (*n* = 153), a woman with a smoking male partner had lower ICSI success rates (22 %), compared to 8 % for women with non-smoking partners [23]. Furthermore, the authors found similar results for IVF, where IVF success rate was 18 % for women with smoking partners and 32 % with non-smoking partners in 213 treatment cycles. Recent reports have suggested that smoking negatively affects semen quality parameters including morphology [26, 27] and motility [27], yet the biological mechanism remains unclear. However, one of the main constituents of cigarette smoke is the highly mutagenic and carcinogenic compound benzo[a]pyrene [28]. Research has found that sperm exposed to benzo[a]pyrene display a moderately increased amount of DNA fragmentation, along with increased benzo[a]pyrene adducts on DNA in comparison with non-exposed sperm [29]. Sperm DNA damage is associated with an increased risk of pregnancy loss after IVF [30], therefore exposure to benzo[a]pyrene and the resulting DNA damage could be a potential mechanism leading to increased risk for pregnancy loss with male smoking partners. Taken together, all of these results, including the previously unpublished results from our laboratory, imply that for women undergoing IVF treatment with a partner who smokes cigarettes, there may be a negative impact on the subsequent IVF outcome. Consequently, couples undergoing IVF treatment should consider the cessation of smoking to enhance likelihood of a successful IVF outcome.

### Alcohol consumption

Alcohol consumption in women has been associated with decreased fertility [3] and decreased chance of conception [31]. In a study involving 7393 women over an 18 year period, the women who were either low or high consumers of alcohol were shown to have given birth significantly fewer times compared to women who were moderate alcohol consumers [3]. However, the authors did not find any difference in miscarriage rate or extra-uterine pregnancy rates between the low and high consumers of alcohol [3].

In 1998, another study focused on the effects of alcohol consumption on fecundability [31]. They observed that during the 6 cycles of follow up, women who consumed no more than five alcoholic drinks per week had achieved higher conception (64 %), than women with a higher alcohol intake (55 %). Similarly, women who consume more than seven alcoholic drinks per week were shown to take a significantly longer time to become pregnant compared with those who consume less alcohol per week [32]. Conversely, no statistically significant relationship between alcohol consumption in males and onset of pregnancy in their partners was observed [32]. Moreover, women who consume over 5 units of alcohol per week are also more likely to experience a spontaneous abortion compared with those who consume no alcohol [33].

Male fertility is also affected by consumption of alcohol and a recent study demonstrated that infertile men classified as daily drinkers, have significantly inferior semen quality (sperm analysis parameters) and hormonal characteristics compared to occasional drinkers [34]. Other research suggests that alcohol consumption also impacts on sperm morphology and sperm count. Another study found that heavy alcohol consumers showed higher cases of teratozoospermia and oligozoospermia when evaluated against men who do not consume alcohol [4]. In addition, a meta-analysis included 57 studies and found that alcohol consumption led to a significantly reduced semen volume [35]. Other reports have suggested that some of the sperm deterioration in males caused by alcohol consumption may be partially reversible if the patient stops drinking alcohol, although further research is required [36]. While most studies suggest a relationship between alcohol and male fertility, a recent study found no significant relationship between alcohol and sperm parameters including sperm count, sperm motility and morphology in a sample of 121 fertile men, and a group of 42 subfertile asthenozoospermic men [37].

### Alcohol and IVF

#### PIVET data demonstrating the influence of alcohol consumption on IVF outcomes

The effect of alcohol consumption on reproductive outcomes has been documented in several studies over recent years [37–39]. Although most work suggest an adverse relationship between alcohol consumption and reproductive outcomes [38, 39], some studies were not able to identify a direct relationship [37]. Consequently, the true impact of alcohol on IVF outcomes is complex and not entirely clear. Considering that alcohol consumption is a common lifestyle factor among many individuals, the effect of alcohol consumption on IVF outcomes needs to be determined. The data obtained from our laboratory demonstrated no evidence of a significant relationship between female alcohol consumption and fertility parameters such as, oocyte production, fertilization rate, β-hCG pregnancy rate or pregnancy loss (Tables 2 & 3). The outcomes were the same even after adjusting for several variables like patient age, BMI and oocyte quality. This could be because females tended to decrease their alcohol consumption before undergoing IVF treatment in order to increase their chance of a pregnancy.

Conversely, we observed that male alcohol consumption had a positive effect on fertilization rate (β = 0.074, *P* < 0.001) (Table 2) and did not impact significantly on first-trimester pregnancy loss (Table 3). The cause of this positive effect on fertilization is difficult to determine. However, we speculate that consumption of fruits and vegetables, which is also included in our work, may improve rates of fertilization, and our results suggested that there was a significant interaction between these two variables (Table 2). Furthermore, this indicated that a common factor derived from both may promote the observed positive effects on fertilization.

From a nutritional perspective, one potential common compound that may mediate these responses is folic acid. Alcoholic beverages such as beer, has a high folate content, and studies have revealed that there is a positive correlation between beer intake and blood folate [40]. In addition, dark leafy green vegetables, such as avocados, broccoli and asparagus, also have high folate content. Interestingly, low folate concentration in seminal plasma has been correlated with increased sperm DNA damage [41] and folate is known to have antioxidant properties that may counteract negative effects of ROS (Fig. 1) [42], which may in turn be one of the possible reasons for the positive effects of folate-containing food and beverages. The impact of folate on IVF outcomes is currently under consideration by our group.

#### Review of data from the literature demonstrating the influence of alcohol consumption on IVF outcomes

A recent large study in 2545 couples demonstrated that following adjustment for age, BMI and cigarette use, women who consumed four or more alcoholic drinks per week (*n* = 237) had a 16 % lower chance of live birth in comparison to women consuming less (*n* = 994) [38]. For couples where both members consumed more than 4 alcoholic drinks, there was a 21 % lower chance compared with couples who consumed less [38]. This was contradictory to our results, which showed no evidence of a significant relationship between female alcohol consumption and pregnancy or pregnancy loss, but may be explained by the difference in sample size and population (*n* = 2545 versus *n* = 68; Table 3). However, they also demonstrated that the overall effect of male alcohol consumption and pregnancy loss in their partners was not significant and this was similar to our findings [38]. On the other hand, associations were found between alcohol consumption and fertilization. In men and women consuming four or more alcoholic drinks per week, there was a 48 % decreased chance of fertilization [38], but this was not observed in our study (Table 2).

Given the controversial beneficial effects of male alcohol consumption on IVF outcomes shown here, it is not surprising that other studies have failed to reveal any beneficial effect, and this further complicates the outlook in relation to alcohol consumption and IVF. Klonoff-Cohen et al. [39], demonstrated that alcohol consumption was associated with a 13 % decrease in number of oocytes retrieved, even after adjusting for other confounding factors such as age, race, reason for infertility and smoking status in 221 couples with female infertility. Female consumption of alcohol one month and one week before the procedure date increased the risk of not becoming pregnant in comparison to controls (women who did not consume alcohol). Moreover, there was a trend towards an increased risk of miscarriage in women who consumed alcohol one week before the procedure. However, similar to our results, there was no significant association between female alcohol consumption and live birth rates [39]. However, in this study, women who drank 12 g more of alcohol per day during the first week of the attempt, this resulted in a 59 % increase in fertilization. Due to the fact that women undergoing IVF treatment modify their behaviour to increase their chance of IVF success, this could have potentially caused the non-significant effect of alcohol consumption seen in our results. However, in that study only 6 % of the women consumed alcohol during the treatment, while approximately half of the patients in our study consumed alcohol during treatment, although the average consumption rate was less than one standard drink per day. Therefore, women who are highly motivated to have a successful IVF outcome are more likely to drink less alcohol or stop drinking altogether. Also it is important to take into account that most of our data was obtained through patient questionnaires. Patients may over-report behaviours seen as socially desirable and may under-report behaviours they deem not to be.

Interestingly, these researchers reported that male consumption of alcohol did not affect sperm count, motility, morphology, fertilization rate or chance of pregnancy [39]. However, more recent studies suggested that sperm concentration, total count and progressive motility were reduced in alcohol consumers in both IVF and control male patients [26], while DNA damage was increased in sperm derived from infertile man who consumed alcohol [43]. Others have also indicated that alcohol also impacts on semen volume, along with levels of male sex steroids [44], which could impact on IVF outcomes. The impact on DNA damage is discussed in more detail and against the backdrop of reactive oxygen species (ROS) below.

Taken together, these data suggest a possible relationship between alcohol consumption and several IVF outcomes and semen parameters (Fig. 1). Interestingly, the studies described above along with our data constitute some of the only research material in this area, and consequently much more research is required to fully understand the complex relationship between alcohol consumption and IVF outcomes.

## Reactive oxygen species (ROS) and reproduction

### The involvement of ROS in IVF outcomes from lifestyle factors

ROS are produced as by-products of the normal metabolism of oxygen and they include a range of molecules such as hydrogen peroxide (H_2_O_2_), superoxide anion (O_2_), hydroxyl free radical (OH^·^), among others [45]. Excessive production of ROS can cause deleterious effects on cell membranes, DNA and proteins. Consequently, there must be a balance between excessive formation of ROS and antioxidant defences [45]. In turn, oxidative stress is the term used to describe the uncontrolled production of ROS and decreased/impaired antioxidant function [46]. ROS have been found to cause effects on the reproductive system as a result of endogenous synthesis. However, lifestyle choices such as smoking, chronic alcohol consumption, and poor nutritional habits (i.e. low fruits and vegetables, high lipids and sugar), potentiate the endogenous production of ROS and the exogenous exposure, promoting oxidative stress (Fig. 1) . Since excessive ROS and oxidative stress cause adverse effects on the reproductive system, it is reasonable to assume they also have an impact on IVF outcomes.

### ROS, cigarette smoking and alcohol

Some of the health risks associated with inhaling cigarette smoke are due to the chronic effects of excessive oxidative stress. Cigarette smoke has been found to contain over 4000 chemicals [47], and a smoker can be directly exposed to over 10^15^ ROS per puff in the gas phase of smoking in addition to poisonous cigarette tar [48]. Furthermore, those authors point out that tar, the particulate matter retained on the cigarette filter, has been found to contain polyphenols, while the gas phase smoke has been found to contain high concentrations of nitric oxide (NO). These two phases contain oxidizing chemicals, which exposes the lungs as well as the entire organism to oxidative stresses [49]. Smokers have also been found to have lower circulating concentrations of antioxidants, which may exacerbate rising ROS levels derived from cigarette smoke. Furthermore, alcohol has also been implicated as the source of ROS that contributes to the pathogenesis of alcoholic liver disease, as demonstrated by detection of lipid peroxidation markers in the liver of alcoholic patients [50]. However, given the extent of knowledge related to the consequences of excessive oxidative stress, it is surprising that little research exists examining the impact of ROS derived from alcoholic drinks and cigarettes, on the reproductive system. Consequently, more research needs to be undertaken to fully determine the influence of this source of ROS on fertility.

### ROS and male fertility

Research conducted over the past 25 years has suggested that a possible cause of sperm dysfunction is oxidative stress [51, 52]. Sperm naturally produce different ROS types, including NO, O_2_^·^, and H_2_O_2_ [53, 54]. Even though excessive levels of ROS can have a detrimental effect on sperm quality and therefore male fertility, not all ROS produced by sperm are harmful as low levels of ROS play a physiological role in the regulation of capacitation, hyperactivation and the binding of the sperm to the zona pellucida [55]. On the other hand, there are a few positive effects of ROS on sperm, but when the amount of ROS exceeds the already limited antioxidant defences of sperm, oxidative stress occurs.

Under low-grade chronic oxidative stress-inducing conditions, damage to the sperm plasma membrane and DNA ensues. Research has found that sperm derived from infertile men display damage to plasma membrane proteins leading to loss of plasma membrane function, which leads to altered functions in sperm motility, ability of sperm to bind to the zona pellucida and sperm-oocyte function [56]. Sperm with low motility, DNA fragmentation damage and non-viable sperm produce higher levels of ROS [56]. An Australian study showed that DNA fragmentation in sperm from ART patients is correlated with expression of 8-OHdG (8-oxo-2-deoxyguanosine), a marker for oxidative stress-induced DNA damage [57]. DNA fragmentation is negatively correlated with pregnancy in natural and ART conceptions such as IVF, but not following intracytoplasmic sperm injection (ICSI) [58].

Another study found that sperm DNA fragmentation was negatively correlated with number of quality embryos and pregnancy rate [58]. This suggested there is a relationship between oxidative stress in sperm and pregnancy outcome in IVF patients. As has been previously mentioned, our research found that for male smokers there was an increased risk of first-trimester pregnancy loss (Table 3). Cigarette smoking is recognized as a source of ROS and increased ROS has been found to cause sperm DNA damage, therefore this could be the potential mechanistic cause of the increased risk of pregnancy loss related to male smokers.

### ROS and female fertility

ROS has a deleterious effect on the development of human oocytes, as well as developing embryos [59]. Oxidative stress is thought to be one of the major causes of female fertility problems such as tubal infertility, endometriosis [60] and polycystic ovary syndrome [61]. A study conducted in 2013, found significantly increased levels of ROS in women failing to become pregnant from a wider sample of women undergoing IVF treatment [60]. In addition, they also found that women with higher levels of ROS produced more immature and lower quality embryos. These results suggest that in women undergoing IVF, pregnancy outcome is affected by the presence of oxidative stress in both endometriosis and tubal infertility. The direct effect of ROS on IVF treatment in females has not been examined at length and this is an interesting are of research requiring more attention.

## Nutrition and reproduction

### Lifestyle related to nutrition

Due to the complexity, well designed studies investigating how the diet affects fertility is very limited. However, there are studies that have demonstrated that fertility is decreased in both overweight men and overweight women [62, 63]. Another investigation conducted in 2007 found that those who had an increased energy intake from trans unsaturated fats or trans-fatty acids, as opposed to carbohydrates, experienced a 73 % increased risk of ovulatory infertility [63]. However, the aforementioned study did not include the male partner’s dietary intake, which is therefore a significant limitation. In males, a higher antioxidant intake (vitamin C, vitamin E, folate and zinc) has been suggested to be associated with better semen quality [64]. Specifically, vitamin C was associated with higher sperm count and concentration, while vitamin E was related with progressive motility [64]. These results suggest an increased antioxidant intake may accompany increased sperm motility and numbers. However, in this study, those who had ‘high’ levels of antioxidants as opposed to moderate or low levels, achieved this high level through diet and supplementation as opposed to diet alone (100 % of vitamin C group and 83 % of the vitamin E group) [64]. It would be interesting to know whether these results could be replicated with just a high vitamin C or E diet only.

In relation to sperm DNA damage, studies have found that men with the high intake of vitamin C through diet and supplementation, had around 16 % less sperm DNA damage compared with the lowest intake group [65]. In addition, older men were most likely to benefit from this. Similar findings were found with other antioxidants including, vitamin E, zinc and folate [65] and these findings suggest that antioxidant supplementation could potentially reverse some of the sperm DNA damage caused by oxidative stress. Similar to males, the main nutritional or dietary factor studied in female fertility was vitamins and antioxidants. One study conducted in 2012 found that shorter ‘time to pregnancy’ was achieved with increasing vitamin C, but this effect was only seem in women with a body mass index (BMI) > 25 kg/m2, and in women over 35 [5]. However, again as with the male study above, they stated that the increased vitamin levels is derived from diet and supplementation rather that diet alone.

### Nutrition and IVF

#### PIVET data demonstrating the influence of fruit & vegetable intake on IVF outcomes

The main nutritional factor measured in the study from our laboratory was fruit and vegetable consumption. Intake of fruits and vegetables may be an overall indicator of diet quality, and a diet of high quality most likely provides essential nutrients. In our study, the only IVF outcome found to be significantly influenced by fruit and vegetable consumption was fertilization rate and this was seen in males only, and not in females (Table 2). Male fruit and vegetable consumption had a positive effect on fertilization rate (β = 0.034, *p* < 0.001) (Table 2). Unfortunately, six males never completed the F&V section of the survey even after several attempts to contact them. This accounted for 2 % of the male population. The general response rate of males for F&V was good (98 %), but we speculate that these six individuals may have been in the lower quartile of F&V consumers, and may have not reported because they considered the low intake of F&V to be not socially desirable. In this case, inclusion of their data may have strengthened the logistic regression analysis showing that F&V consumption had a positive effect on fertilisation rates. However, if the opposite was true and they were high F&V consumers with low fertilization rates, it is unlikely that their outcomes would influence the highly significant correlation observed in our analysis (β = 0.034, *p* < 0.001) (Table 2). Fruit and vegetables contain a high level of antioxidants [66], consequently, these antioxidants may have potentially reversed or protected sperm from DNA damage induced by ROS, which subsequently increased fertilization rates. On the other hand, our female population may not have had enough ROS-related disorders to show any significant beneficial effects from the antioxidants contained in fruit and vegetables.

Fruit and vegetable consumption had no significant influence on first-trimester pregnancy loss (Table 3). This was evident for both male and female fruit and vegetable consumption. Consequently, these data indicate that the possible benefits of dietary intake relate to fertility parameters upstream of implantation and may associate with better semen or oocyte quality.

#### Review of data from the literature demonstrating the influence of fruit & vegetable intake on IVF outcomes

Generally, research on the effect of nutrition in IVF outcomes, especially fruit and vegetable intake, is limited. This is an important area of research that needs to be expanded upon given the current trends of increased infertility, particularly against the backdrop of over-nutrition in the existing population. The few studies that have focused on the relationship between nutrition and IVF demonstrate varying results. The associations between serum polyunsaturated fatty acids (PUFAs) and embryo implantation rates in women undergoing IVF were examined recently [67]. PUFAs derived from the diet that were measured included linoleic acid (LA) and alpha-linolenic acid (ALA). Their results found that no specific PUFA was associated with chance of pregnancy, but interestingly the ratio of LA to ALA was significant with regard to pregnancy. When adjusted for confounding factors such as age, endometriosis, peak oestradiol level and male factor fertility, it was found that there was a dose dependent increase in chance of pregnancy with increased LA:ALA ratios, and this was also weakly correlated with embryo implantation rates [67]. Although this study does suggest that supplementation of PUFA’s could possibly improve fertilization rates, further studies are needed to determine if such supplementation could increase pregnancy in couples undergoing IVF treatment.

In addition, an investigation conducted by Vujkovic et al. in 2010 demonstrated the impact of diet on IVF outcomes in 160 couples undergoing IVF/ICSI treatment at a fertility clinic in the Netherlands. They identified two types of dietary patterns in women who participated in the study. A ‘health conscious-low processed’ dietary pattern, which consisted of a high intake of vegetables, fruit, grains whole-grains, and a low intake of snacks, meat and mayonnaise. High adherence to this diet was correlated with an increase in red blood cell folate [68]. The other dietary pattern was identified as the “Mediterranean” diet, which consisted of a high intake of vegetable oils, vegetables, legumes, fish, and a low intake of food snacks or fast foods. High adherence to this diet was correlated with increased folate, vitamin B6 in the blood and follicular fluid. In terms of IVF success, neither diet were associated with embryo quality or fertilization rate, but a high adherence to the Mediterranean diet was associated with a 40 % increase in the probability of pregnancy after IVF/ICSI [68]. Interestingly, these observations were adjusted for confounding factors and were not significantly affected by lifestyle factors of the males including age, alcohol consumption, smoking and BMI. The authors suggested that the reason for the increase in pregnancy chance when adhering to the ‘Mediterranean’ diet was due to the high intake of vegetable oils, which are high in linoleic acid. At the molecular level, linoleic acid is a precursor for prostaglandins, which are important in growth and development of ovarian follicles, the process of ovulation in addition to the development of appropriate endometrial receptivity. Therefore, these results suggested that an increased intake of linoleic acid could be beneficial for implantation of a fertilized embryo. One significant limitation of this latter study, was that the diet of the male partner was not included and as demonstrated in our own results, increased fruit and vegetable consumption in males caused an increase in fertilization rate, ultimately suggesting that the male diet is also an important factor to consider (Table 2).

Interestingly, a similar study from the same group was conducted in males from 161 sub-fertile couples in the Netherlands [69]. Here, they identified two patterns of diets—a “health conscious” diet containing high intake of fruit, vegetables, fish, seafood, legumes and whole-grains, and low intakes of fatty sauces, meat, sugar, confectionary and refined grains, and the “Traditional Dutch” diet, which contained high intakes of potatoes, meat, whole grains, fatty sauces such as mayonnaise, and a low intake of alcohol, cereals, fruit, sugar and soup. They found that the “health conscious” diet was inversely proportional to sperm DNA damage, which was explained by high intakes of fruit and vegetables when examined individually [69]. Furthermore, we speculate that this inverse relationship between high intakes of fruits and vegetables and sperm DNA damage might be due to high levels of antioxidants contained within fruits and vegetables.

Increased antioxidant intake has been associated with high sperm numbers and motility [64] and reduction of the incidence of sperm DNA fragmentation [66]. Participants consuming the “Traditional Dutch” diet, showed increased sperm concentration explained by the low intake of alcohol and high intake of potatoes [69]. These results could explain the relationship between unhealthy diets and decreased semen quality in Westernized countries. However, more studies are required to determine the specific cause of these associations.

Increased fruit and vegetable consumption was associated with increased fertilization rates in our study, suggesting a possible relationship between male diet and some IVF outcome parameters (Table 2). However, as we are one of the few groups to show this relationship, there is a requirement for further research to elucidate precisely how the diet of males affects IVF outcomes in their partners. These studies would help to determine the relationship between IVF success and male diet, which could possibly help increase the success rate of IVF if the male patient knows how to adjust their own diet to accommodate for this. Further research also needs to be undertaken to determine the molecular compounds causing the nutritional effects in IVF, and it is possible that a diet can be designed for both men and women, which will provide the greatest benefit for women undergoing IVF treatment.

## Conclusion

Based on our results and the current literature, there is an important effect of lifestyle factors in mediating IVF clinical outcomes (Fig. 1). Of significance is the finding that there is a risk of increased pregnancy loss with paternal smoking, which is most likely due to spermatozoal DNA damage. Moreover, female smoking has an adverse effect on ovarian reserve and that oocytes exposed to smoking in the past, have decreased fertilization rates. There are conflicting results regarding other lifestyle factors such as nutritional habits and alcohol consumption. It must be highlighted that only 80.1 and 70.4 % of females and males, respectively, returned their one week diary to document their smoking, alcohol and fruit and vegetable intake. Therefore, this level of non-response is not negligible and may introduce some non-response bias, which is a significant limitation of the current study. Nonetheless, it is apparent that chronic oxidative stress induced by lifestyle factors and poor nutritional habits associate with a lower rate of IVF success. Further optimally designed studies, which are appropriately powered, are clearly necessary to precisely correlate the impact of these lifestyle behaviours on fertility and treatment outcomes.
